# Investigating Bubble Formation and Evolution in Vanadium Redox Flow Batteries via Synchrotron X‐Ray Imaging

**DOI:** 10.1002/cssc.202500282

**Published:** 2025-04-24

**Authors:** Kangjun Duan, Kerstin Köble, Alexey Ershov, Monja Schilling, Alexander Rampf, Angelica Cecilia, Tomáš Faragó, Marcus Zuber, Tilo Baumbach, Pang‐chieh Sui, Roswitha Zeis

**Affiliations:** ^1^ Helmholtz Institute Ulm Karlsruhe Institute of Technology 89081 Ulm Germany; ^2^ Laboratory for Applications of Synchrotron Radiation Karlsruhe Institute of Technology 76131 Karlsruhe Germany; ^3^ Institute for Photon Science and Synchrotron Radiation Karlsruhe Institute of Technology 76344 Eggenstein‐Leopoldshafen Germany; ^4^ School of Automotive Engineering Wuhan University of Technology Wuhan 430070 China; ^5^ Institute for Integrated Energy Systems University of Victoria BC V8W 2Y2 Canada; ^6^ Friedrich‐Alexander Universität Erlangen‐Nürnberg (FAU) Faculty of Engineering, Department of Electrical Engineering 91058 Erlangen Germany; ^7^ Department of Mechanical and Industrial Engineering University of Toronto Toronto Ontario M5S 3G8 Canada

**Keywords:** bubble analysis, hydrogen evolution reactions, synchrotron X‐ray imaging, vanadium redox flow batteries

## Abstract

The parasitic hydrogen evolution reaction (HER) hinders electrolyte transport. It reduces the effective electrochemical surface area in the negative half‐cell of vanadium redox flow batteries (VRFBs), resulting in substantial efficiency losses. This study investigates the formation and evolution of hydrogen bubbles within VRFB electrodes through comprehensive experimental characterization and a detailed analysis of the resolved bubbles. The electrode is imaged using synchrotron X‐ray tomography, and gas bubbles in the images are identified and characterized using a deep learning model combined with a morphological analysis tool. The HER intensity increases at more negative working electrode potentials, causing residual bubbles to grow and fuse in the electrode central region. In contrast, independent bubbles predominantly form at the electrode edges. Furthermore, bubble growth leads to the gradual development of irregular shapes. These observations provide insights into bubble formation and evolution rules, contributing to a better understanding of the system.

## Graphical abstract

This study investigates bubble evolution in vanadium redox flow batteries (VRFBs) under different applied potentials using synchrotron X‐ray tomography. The workflow includes image processing, 3D reconstruction, manual annotation, automated segmentation via deep learning, and bubble characterization. The analysis provides quantitative insights into bubble dynamics, contributing to a deeper understanding of gas evolution and transport in VRFBs.

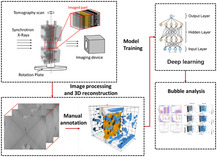

## 1 Introduction

With global warming becoming more severe, the interest in renewable and sustainable energy has grown globally. However, these so‐called green energy sources are naturally subject to fluctuations in power output.^[^ ^1^, ^2^, ^3^, ^4^ ^]^ Because of its power and energy decoupling, potential low cost, fast response, and flexible design, a vanadium redox flow battery (VRFB) can meet the needs of these new energy sources for intermittent or long‐term energy storage.^[^ ^5^ ^]^ The VRFB system is relatively simple compared to other energy storage systems and relies on the reversible redox reactions of vanadium ions, which only change their valence state, thereby avoiding solid‐state reactions.^[^ ^6^ ^]^ This characteristic can theoretically lead to an extended cycle life, although side reactions still pose limitations in practice. In the operating potential window of VRFBs, side reactions cause various issues, including reduced battery efficiency, material corrosion, and gas generation and accumulation.^[^ ^4^, ^7^, ^8^, ^9^, ^10^ ^]^ Here, we focus on the negative half‐cell, in which vanadium(III) is reduced to vanadium(II) during charging. Due to the applied potentials in this half‐cell, hydrogen evolution is triggered as a side reaction. Therefore, this undesired side reaction consumes charges unavailable for the desired vanadium reduction reaction. Thus, electrolyte imbalance occurs, and the battery capacity and coulombic efficiency fade over time.

Existing research on hydrogen evolution reaction (HER) in VRFBs has primarily focused on elucidating the fundamental reaction mechanisms at the electrode–electrolyte interface. Various strategies have been developed to mitigate HER in VRFBs. For example, researchers have improved the catalytic properties of carbon electrodes through thermal treatment, doping, or surface functionalization to suppress HER.^[^ ^11^, ^12^ ^]^ In addition, altering the interfacial properties by introducing surfactants or other organic compounds into the electrolyte has been shown to reduce HER, although such additives can sometimes compromise long‐term stability.^[^ ^13^, ^14^ ^]^ Alternatively, operating the negative electrode within a more restricted potential window can limit HER, but this approach may also reduce the efficiency of the desired redox reactions.^[^ ^15^ ^]^ While these approaches have demonstrated efficacy in mitigating HER and improving macroscopic battery performance, they generally do not provide detailed insights into the microscopic processes governing bubble formation and evolution within the electrode. Recent studies have begun to explore the microstructural characteristics of gas bubbles in porous electrodes. For example, Bevilacqua et al. used synchrotron X‐ray tomography to investigate the impact of compression ratio on the electrolyte distribution. They found that remaining air bubbles in the carbon felt material and compression ratios significantly influence electrolyte saturation.^[^ ^16^ ^]^ Li et al. conducted in situ experiments to investigate the HER in a transparent cell. They observed a sequential process with the generated hydrogen gas adsorbing onto the electrode surface and migrating, forming larger bubble agglomerations. This phenomenon, driven by buoyancy, ultimately blocks the transport pathway of the electrolyte.^[^ ^17^ ^]^ Eifert et al. presented an innovative experimental setup for VRFB that enabled synchrotron X‐ray imaging under potential or current control. They observed an overall decrease in the average saturation during the redox cycles owing to side reactions.^[^ ^18^ ^]^ Köble et al. utilized synchrotron radiation‐based X‐ray imaging techniques to visualize bubble distribution within carbon‐felt electrodes under varying negative working electrode potentials.^[^ ^19^ ^]^ Their findings reveal that bubble formation is predominantly localized at the borders and cutting edges of electrodes, serving as preferred nucleation sites due to material heterogeneities. Furthermore, the study quantifies the trend of increased bubble formation with more negative working electrode potentials, highlighting the need for strategies to mitigate HER activity and its consequences. Zhang et al. investigated the impact of in situ hydrogen evolution on the flow resistance of electrolyte flowing through a carbon felt electrode in a redox flow battery.^[^ ^20^ ^]^ Their work quantitatively demonstrated that even a low level of HER can significantly reduce the relative permeability of the electrode, thereby hindering electrolyte transport. Similarly, Ye et al. revealed a vicious cycle between bubble trapping and flow choking in redox flow battery stacks, showing that the accumulation of gas bubbles not only narrows the liquid flow paths but also leads to further reduction in liquid velocity, exacerbating nonuniform electrolyte distribution.^[^ ^21^ ^]^ Among the various investigations related to bubbles within VRFB electrodes, no research study has yet investigated the individual bubble morphologies. Since the presence of bubbles directly impacts the electrolyte's transport pathway, it is just as essential to study their size and shape as it is to investigate the structure and morphology of the electrode pores.

In other research areas focusing on pore‐scale morphology, a wealth of existing studies provides valuable insights and ideas for our task. Hoehl et al. conducted operando experiments using synchrotron X‐ray radiography to investigate proton exchange membrane (PEM) water electrolysis cells.^[^ ^22^ ^]^ They focused on analyzing the frequency and volume of gas bubbles released from the porous transport layer (PTL) into the flow channels. The study revealed that a water‐saturated PTL exhibited selective transport pathways for the evolved gas, with more pathways occurring at higher current densities. Panchenko et al. employed in‐plane synchrotron radiography to investigate transport phenomena in a PEM electrolysis cell.^[^ ^23^ ^]^ Their study successfully visualized individual bubbles occupying the pores and propagating through the PTL, along with the gas flow from the cathode catalyst layer to the flow channels. Jo et al. employed image processing techniques to investigate the bubble mechanisms within a 2D‐packed bed at the pore level.^[^ ^24^ ^]^ Through an extensive analysis of a substantial collection of images, they identified two bubble coalescence mechanisms and three breakup mechanisms. Furthermore, they derived valuable data on numerous two‐phase parameters, including local void fraction, bubble velocity, size, number, and shape. These studies on bubble dynamics are highly intriguing because they provide critical insights into characterizing and analyzing bubbles. Investigating bubble morphology at various stages offers a valuable understanding of bubble formation and growth mechanisms within the electrode.

In this article, we employ high‐resolution synchrotron X‐ray tomography in combination with deep learning‐based image segmentation to systematically analyze the gas bubbles within VRFB electrodes.^[^ ^17^, ^22^ ^]^ We characterized the distribution, size, and morphology of the formed hydrogen bubbles and elaborated on the impact of the working electrode potential on bubble formation and evolution. Unlike traditional strategies that directly inhibit HER through electrode modification, electrolyte additives, or potential regulation, our work provides a novel, microscopic perspective by quantitatively characterizing bubble morphology. This approach lays the groundwork for future studies to establish quantitative correlations between bubble morphology, electrode microstructure, mass transport, and electrochemical performance. Ultimately, the data presented here will offer new insights for optimizing battery design and mitigating the negative impact of HER.

## 2 Results and Discussion

### 2.1 Detailed Bubble Analysis

This section focuses on one experiment for a detailed bubble analysis, which includes the bubble size distribution and characteristic shape descriptions such as bubble roundness, flatness, and elongation. During the experiment, a working electrode potential of −200 mV was applied when electrolyte flow was stopped. Thus, surface tension and buoyancy only affected the bubble growth and movement processes.

**Figure 1:**
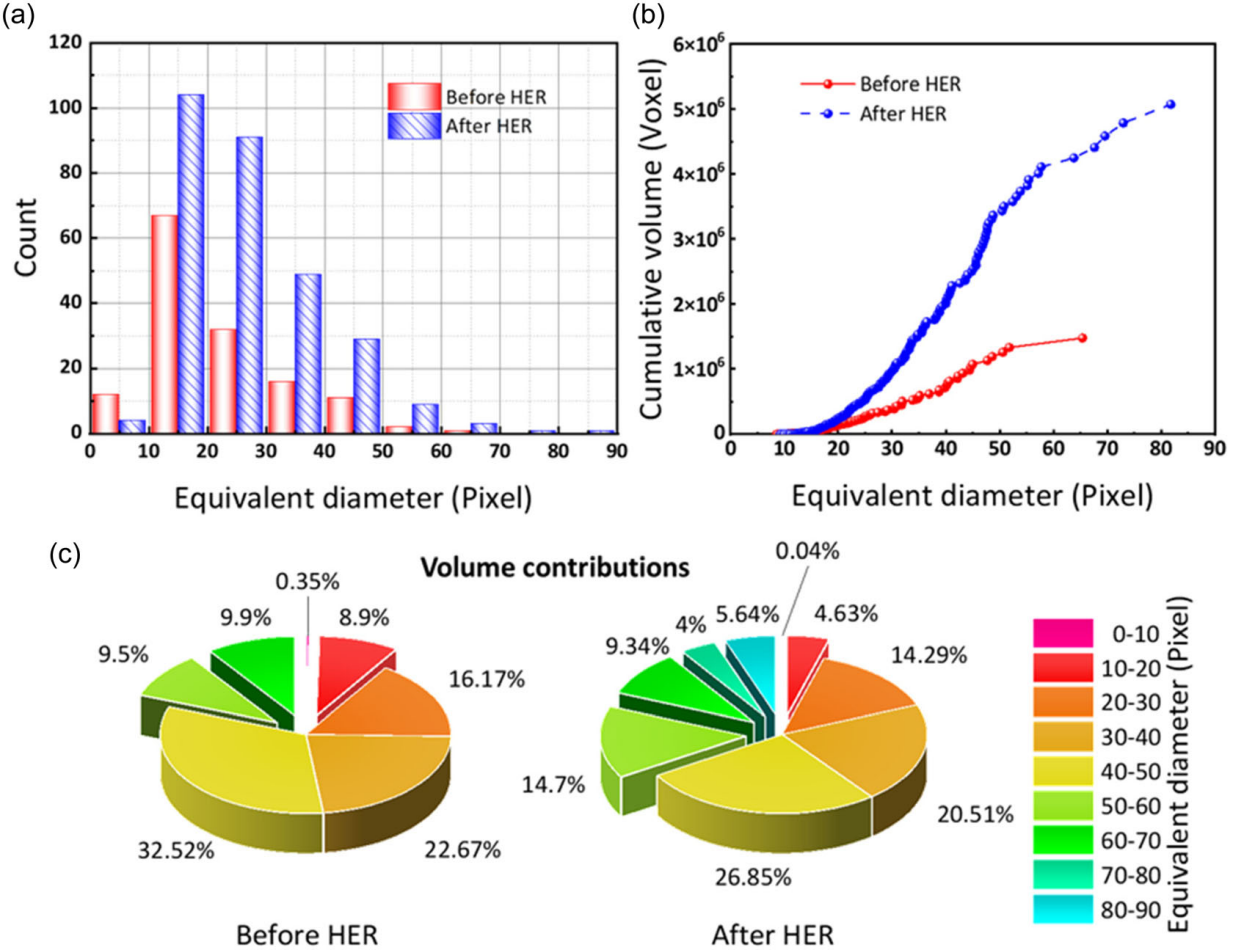
Comparison of the bubble sizes and their contribution to the cumulative bubble volume before and after the HER period at −200 mV: a) size histograms, b) cumulative bubble volumes as a function of equivalent diameter, and c) volume contributions of different bubble sizes.

At the electrode–electrolyte interface, HER produces gas molecules that initially form nanobubble nuclei via homogeneous or heterogeneous nucleation. According to classical nucleation theory, these nuclei arise when the local supersaturation of hydrogen overcomes the energy barrier imposed by surface tension. As the HER proceeds, these nanobubbles grow by absorbing additional hydrogen molecules—a process governed by diffusion‐limited growth—and are simultaneously subject to buoyancy forces that promote their detachment and upward migration.^[^ ^25^ ^]^ However, in a static system, the absence of convective mixing means that coalescence becomes the dominant growth mechanism. When adjacent bubbles come into contact, capillary forces drive their merging, and the resulting bubble's shape is influenced by both the viscous resistance of the electrolyte and the anisotropic microstructure of the electrode fibers. **Figure** **1** presents how the bubble size evolves from the initial electrolyte filling after the applied negative potential triggered the HER. In this study, the size intervals in all histograms are set as 10 pixels. As shown in Figure 1a, most bubbles in the initial distribution have an equivalent diameter of 10−20 pixels, corresponding to a 90−180 μm size. A few bubbles with equivalent diameters smaller than 10 pixels were observed, but the number of bubbles gradually decreased with sizes larger than 20 pixels, and the largest bubble is 66 pixels in equivalent diameter, corresponding to around 600 μm. This observation is consistent with diffusion‐limited growth theory, where bubbles rapidly absorb additional gas and coalesce, leading to a marked shift in the bubble size distribution. After the HER, the number of bubbles with an equivalent diameter below 10 pixels (90 μm) decreases, while the number of bubbles with an equivalent diameter greater than 10 pixels increases significantly. Most bubbles are still observed in the equivalent diameter range of 10−20 pixels, but the largest bubble has an equivalent diameter of 82 pixels corresponding to around 740 μm. A comparison with Figure S1a, Supporting Information, reveals that at −300 mV, the HER is significantly more intense, producing more large‐sized bubbles. At this higher potential, the nucleation rate is so high that small, independent bubbles quickly grow and merge, resulting in fewer overall bubbles but a greater prevalence of super‐sized bubbles. Such behavior aligns with theoretical predictions: As the applied potential becomes more negative, the rate of hydrogen production increases exponentially (per the Tafel equation), and bubble growth becomes more pronounced due to the rapid accumulation of gas molecules. Figure 1b displays the cumulative bubble volume as a function of increasing equivalent diameter, with each data point representing one bubble in the overall volume. It can be concluded that the overall cumulative bubble volume increases quite distinctly from 1.48 × 106 voxels before the HER to 5.08 × 10^6^ voxels afterward. Furthermore, in both cases, there is a sharp increase in the cumulative bubble volume for bubbles in the equivalent diameter range of 20−50 pixels (180−450 μm). From a theoretical standpoint, this sharp increase indicates a transition point where diffusion‐limited growth and bubble coalescence accelerate, leading to a significant volume contribution from bubbles within this size range. The pie charts in Figure 1c further illustrate that bubbles with diameters under 20 pixels are negligible in terms of volume—likely because they represent early‐stage nuclei that either grow or merge—whereas bubbles in the 20–50 pixel range dominate the cumulative volume.

As previously reported, bubbles in VRFB electrodes are unavoidable in the operated potential range if the state of charge is not strongly limited.^[^ ^26^ ^]^ Due to the electrode microstructure, some bubbles are entirely trapped in the electrode, blocking the pores for electrolyte transport. Studying their shape and distribution in the VRFB electrodes is meaningful to optimizing the electrolyte transport process. Thus, this study characterizes the bubbles’ characteristic parameters in detail using the bubble statistical analysis tool library.

**Figure 2:**
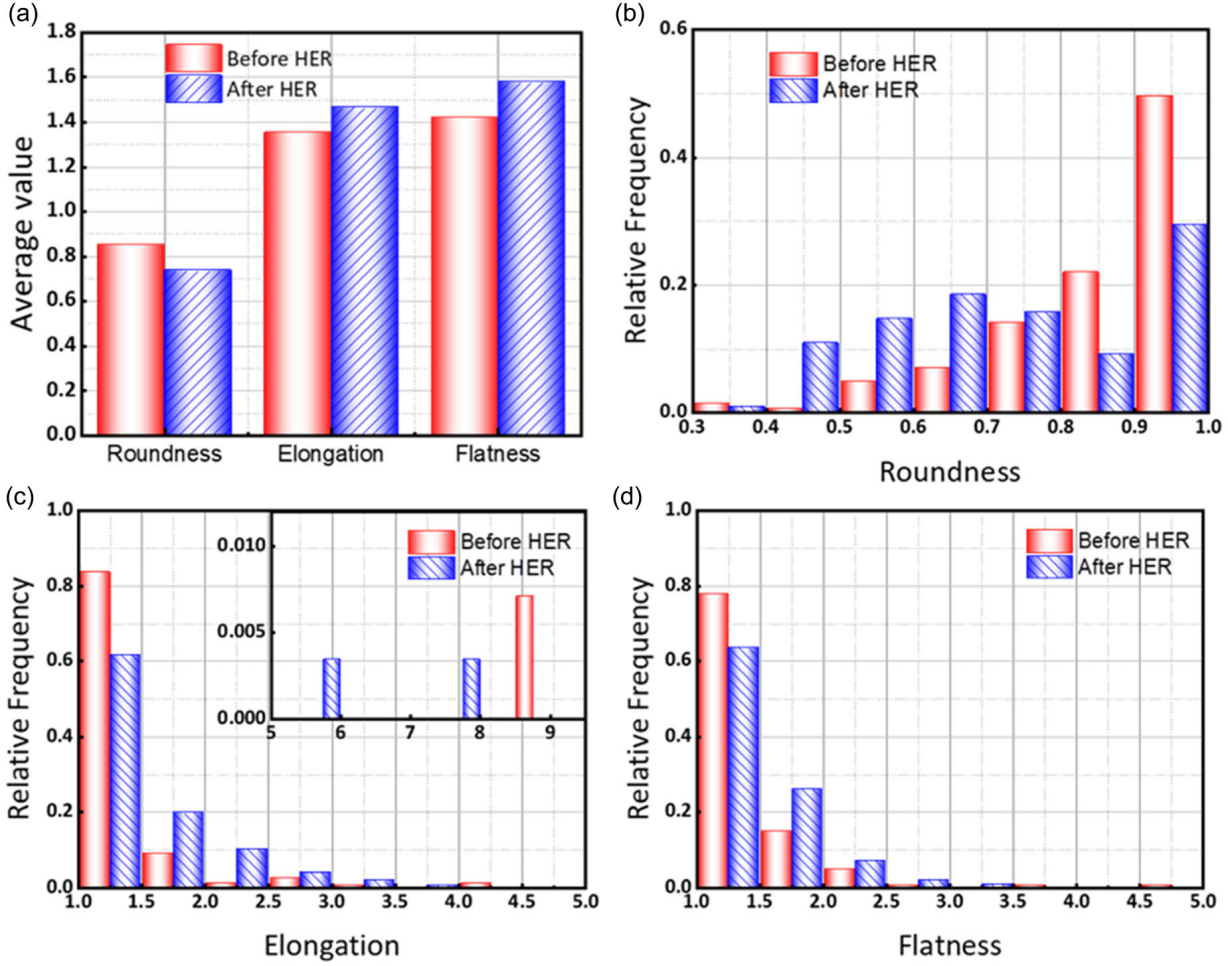
Comparison of the bubble shape statistics before (red) and after (blue) the HER period at −200 mV: a) average shape characteristics, b) roundness histograms, c) elongation histograms, and d) flatness histograms.

The evolution in bubble shape can be understood through the interplay of surface tension, buoyancy, and viscous forces within the electrode's heterogeneous microstructure. **Figure** **2** displays how the bubble shapes change from the initial bubble distribution to after the HER period. For the roundness distribution histogram, the interval is set to 0.1, while the elongation and flatness distribution histograms have intervals of 0.5. The selection of interval sizes is based on their value range. Before HER, most residual bubbles are nearly spherical (with an average roundness of ≈0.83) due to the dominance of surface tension, which tends to minimize the surface area. However, once HER is initiated, rapid bubble growth and frequent coalescence introduce distortions; the bubbles no longer have sufficient time to relax into a spherical shape. After HER, the average roundness in the electrode decreased by 13%, and average elongation and flatness increased by 8% and 11%, respectively, as shown in Figure 2a. This change in shape is theoretically expected when bubbles coalesce in confined or anisotropic media, where spatial constraints force bubbles into irregular configurations. The observation, that the average flatness is slightly higher than the average elongation in both cases, may be explained by anisotropic stress distributions in the electrode structure, which induce preferential deformation along certain axes.^[^ ^27^ ^]^ Notably, in Figure 2b, the roundness frequency distribution is increased within the 0.6–0.7 interval, which is close to the average roundness. The frequency distribution for both elongation and flatness distributions (Figure 2c,d) shifts toward higher values after the HER. However, some highly elongated bubbles can be observed in the elongation distribution before and after the HER. Such bubbles often appear in the edge areas of the electrodes, where the electrode is in contact with other components, such as the flow field, the gasket, or the frame.

**Figure 3:**
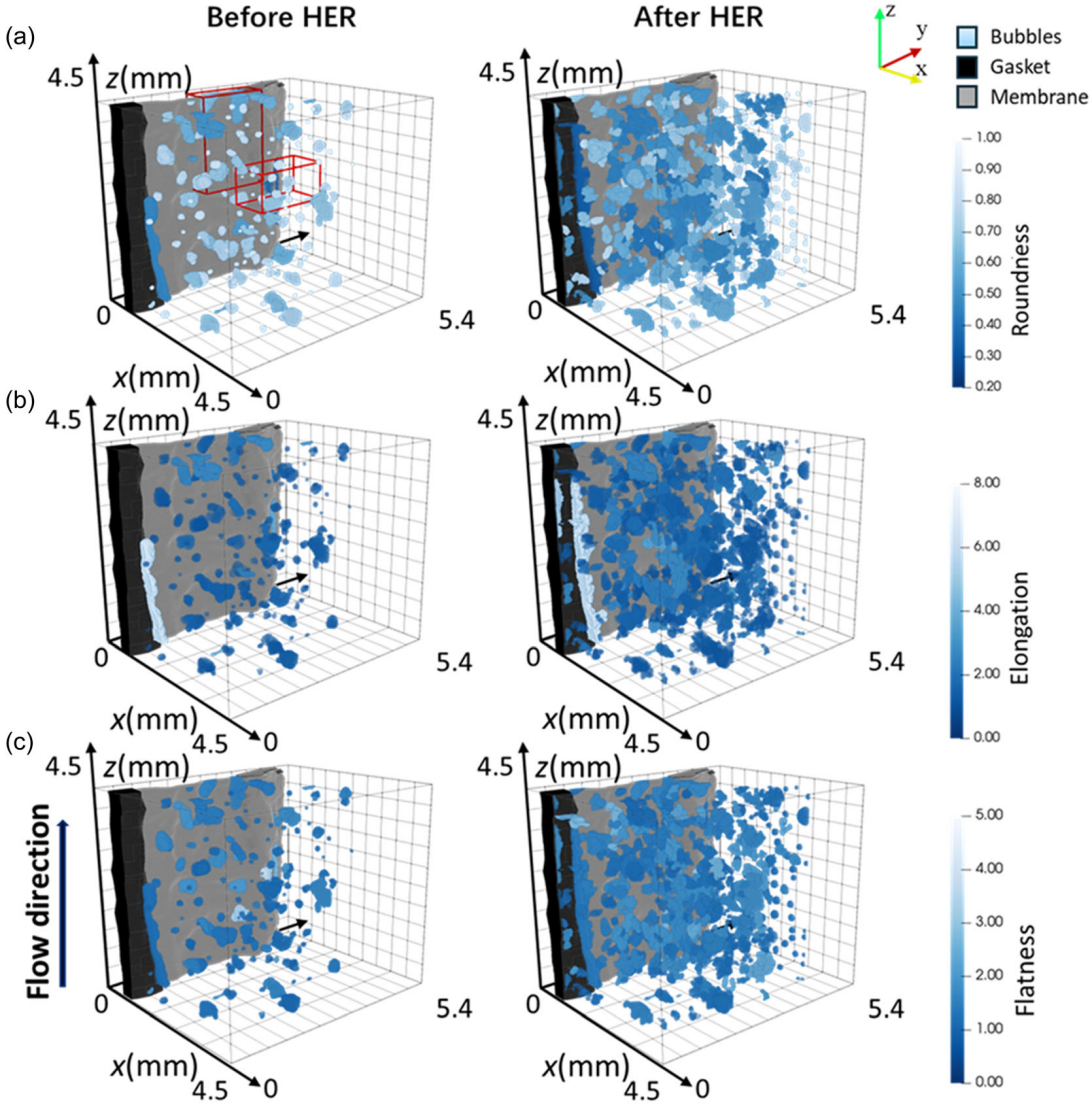
Comparison of the 3D visualization distributions of the bubble shape characteristics for the experiment at −200 mV before and after the HER: a) roundness distribution, b) elongation distribution, and c) flatness distribution.

To relate these observed shape characteristics to specific bubbles and their position within the electrode volume, **Figure** **3** displays the 3D visualized bubbles color‐rendered according to their shape characteristics, further linking the observed morphology to the underlying electrode structure. Figure 3a highlights the different bubble roundness values, whereas Figure 3b shows the elongation variations, and Figure 3c displays the differences in flatness values. Before the HER, the electrolyte refill removed most bubbles near the edges, while bubbles remained in the electrode's center. This may be caused by the inhomogeneous porosity distribution of the electrode due to uneven assembly stress, which is observed by the extrusion deformation of the membrane (Figure S4, Supporting Information). Compared to the measurement at −300 mV (Figure S3 in the Supporting Information), significantly fewer residual bubbles were left in the electrode, especially in the center area.

**Figure 4:**
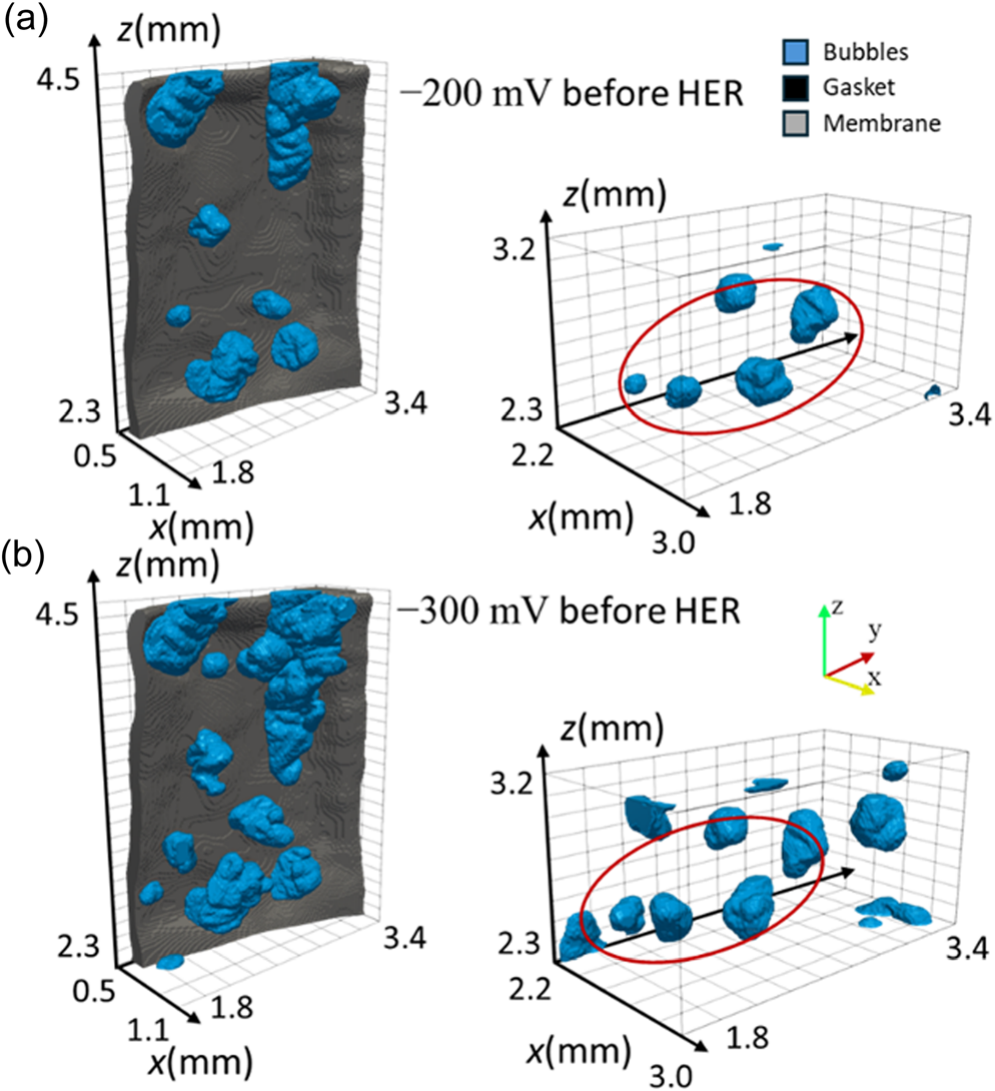
Comparison of local residual bubbles in the marked region of Figure 3a: for the experiment at a) −200 mV and b) −300 mV before HER.

**Figure** **4** compares the local bubble distribution in the red box area in Figure 3a with that of −300mv before HER. We can also observe that the positions of the residual bubbles in the two cases are very similar, and many bubbles with similar shapes can be detected at the same positions. This implies that the electrode microstructure must be highly critical for trapping bubbles, and these fixed positions are extremely unfavorable concerning bubble evolution and trapping. The spherical bubbles with high roundness are mainly distributed in the electrode's center, while the bubbles with high elongation and flatness are observed in the edge area. Furthermore, the rounder bubbles are generally smaller, which shows that the irregular shapes may be a phenomenon related to bubble growth in the inhomogeneous fiber structure. A notable trend emerged when the HER period was completed: Many bubbles with high roundness were generated in the electrode's edge region. In contrast, the bubbles in the center grew larger and became more elongated and flatter. This phenomenon can be attributed to the scarcity of residual bubbles in the edge area, facilitating the formation of new independent and round bubbles. Conversely, the abundance of residual bubbles in the electrode center encourages bubble growth and coalescence. Thus, forming new independent bubbles is notably challenging, and decreased bubble roundness is witnessed in this region.

Overall, the experimental observations are consistent with established theories of nucleation, diffusion‐limited growth, and bubble coalescence. These theoretical considerations not only help explain the statistical distributions of bubble size and shape observed in our experiments but also underscore the critical role of the electrode microstructure in governing bubble dynamics and, ultimately, electrolyte transport.

### 2.2 Effect of Applied Electrode Potential

**Figure 5:**
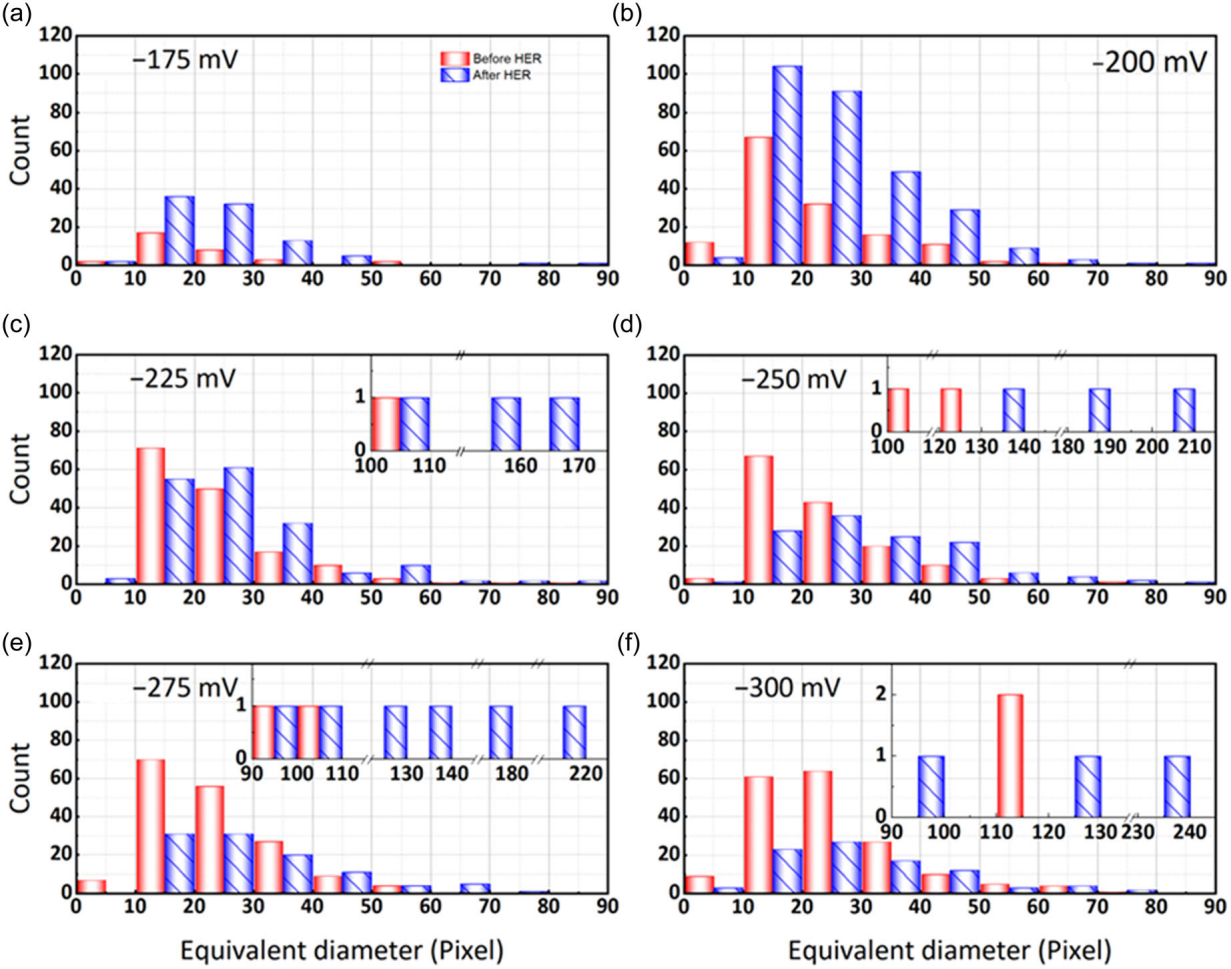
Histograms of the bubble size distributions before and after the HER period at various working electrode potentials: a) −175 mV; b) −200 mV; c) −225 mV; d) −250 mV; e) −275 mV; f) −300 mV.

**Figure** **5** presents the bubble size distribution in the working electrode before and after the HER triggered by applying various electrode potentials between −175 and −300 mV. As investigated in a recent study, the bubble formation at less negative working electrode potentials was low and constant, and significant HER activity was only observed at more negative potentials.^[^ ^19^ ^]^ The bubble size distribution shows a similar trend at each working electrode potential. In all instances, the interval with the highest bubble count consistently falls within an equivalent diameter range of 10−30 pixels (90−270 μm), whether before or after the HER. When the applied potential is −175 mV (Figure 5a), the overall bubble count is significantly lower than in the other experiments, which can be related to the lower HER activity at this potential. At the lower electrode potential of −200 mV (Figure 5b), the most independent bubbles are detected after the HER, which implies that a significant amount of new tiny bubbles evolved at this potential. With the applied potential becoming more negative, the number of bubbles after the HER period gradually decreases, while more bubbles with larger sizes appear. For example, the largest bubble before the HER at −250 mV (Figure 5d) has an equivalent diameter of 123 pixels (around 1100 μm). However, the largest bubble with an equivalent diameter of 201 pixels (around 1800 μm) was detected after the HER. Moreover, fewer bubbles with sizes below 30 pixels (90 μm) are observed after the HER period, which shows that these bubbles must have grown or combined into bigger bubbles. Similar observations were made at more negative potentials of −275 (Figure 5e) or −300 mV (Figure 5f), displaying fewer smaller bubbles since larger bubbles are formed. Thus, these bubble size distributions can provide information on the amount of independently generated bubbles and show the bubble growth and coalescence at different applied electrode potentials.

**Figure 6:**
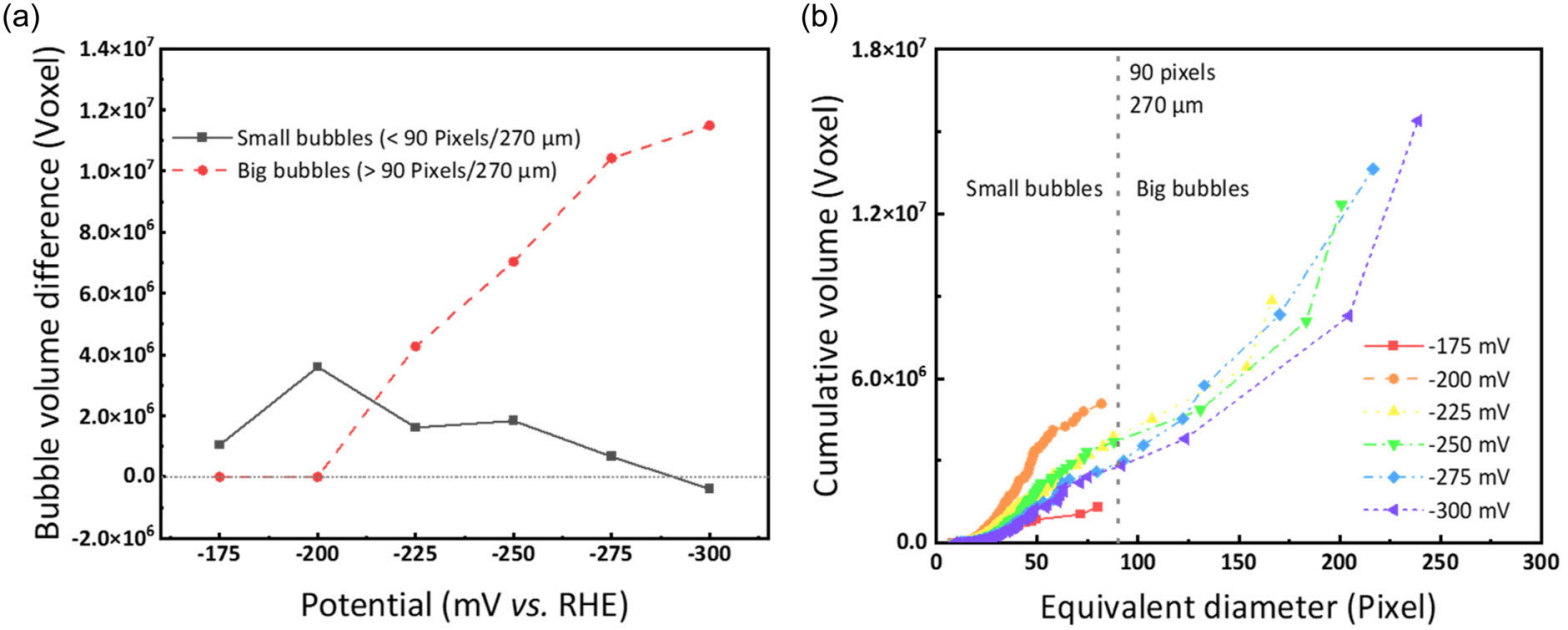
Potential‐dependent bubble volume changes resolving a) the bubble volume differences of small and big bubbles (threshold value: 90 pixels/270 μm) caused by the HER and b) the cumulative bubble volume after the HER as a function of equivalent diameter.

A previous publication from our group already showed that the bubble volume fraction within the electrode volume increases if more negative potentials are applied to the working electrode (Figure S5, Supporting Information).^[^ ^19^ ^]^ This observation supports the knowledge of increased HER activity at more negative electrode potentials. **Figure** **6** presents two ways to resolve the influence of the potential on the bubbles’ sizes. Figure 6a divides the bubbles into two classes based on their size, distinguishing between small and big bubbles. The critical equivalent diameter of 90 pixels (270 μm) for the size‐related classing was defined by the maximum bubble size achieved after the HER at −175 mV (Figure 5a). Based on this categorization, Figure 6a illustrates the volume differences between big and small bubbles during the HER period, subtracting the bubble volume before the HER from the bubble volume after the HER for both size classes. In all experiments, the residual bubbles before the HER are smaller than 270 μm and, therefore, belong to the class of small bubbles. When the applied potential is more negative than −200 mV, big bubbles appear whose equivalent diameters are larger than 90 pixels (270 μm) and whose influence on the cumulative bubble volume change is significantly more significant than small bubbles. At an applied potential of −300 mV, the volume of small bubbles decreased after the HER, implying that the volume of small bubbles generated by the HER is less than that of small residual bubbles merged into big bubbles.

Figure 6b illustrates the cumulative volume added up across different bubble sizes for the experiments at different working electrode potentials. The measurements at −175 and −200 mV stand out from the other applied potentials. At −175 mV, the overall bubble evolution is quite low, as shown in Figure 5a. Thus, the cumulative bubble volume remains low, and small bubbles are generated mainly during the HER period. We already concluded that the measurement at −200 mV shows the highest amount of freshly generated small bubbles which is also evident in Figure 6a. The cumulative bubble volume increases rapidly and gradually but only up to a maximum bubble size of 82 pixels (around 740 μm), and no larger bubbles are generated. At more negative potential, the slope of the cumulative bubble volume decreases, implying that smaller bubbles are detected after the HER. The residual bubbles before the HER grow or accumulate into larger bubbles, leading to a sharp rise in cumulative volume at larger bubble sizes. Such extreme bubbles are generally located at the corners of the electrode and are often generated by the fusion with residual bubbles from the central area. Readers can refer to the Figure S3 in Supporting Information showing the three‐dimensional bubble distribution at −300 mV.

**Figure 7:**
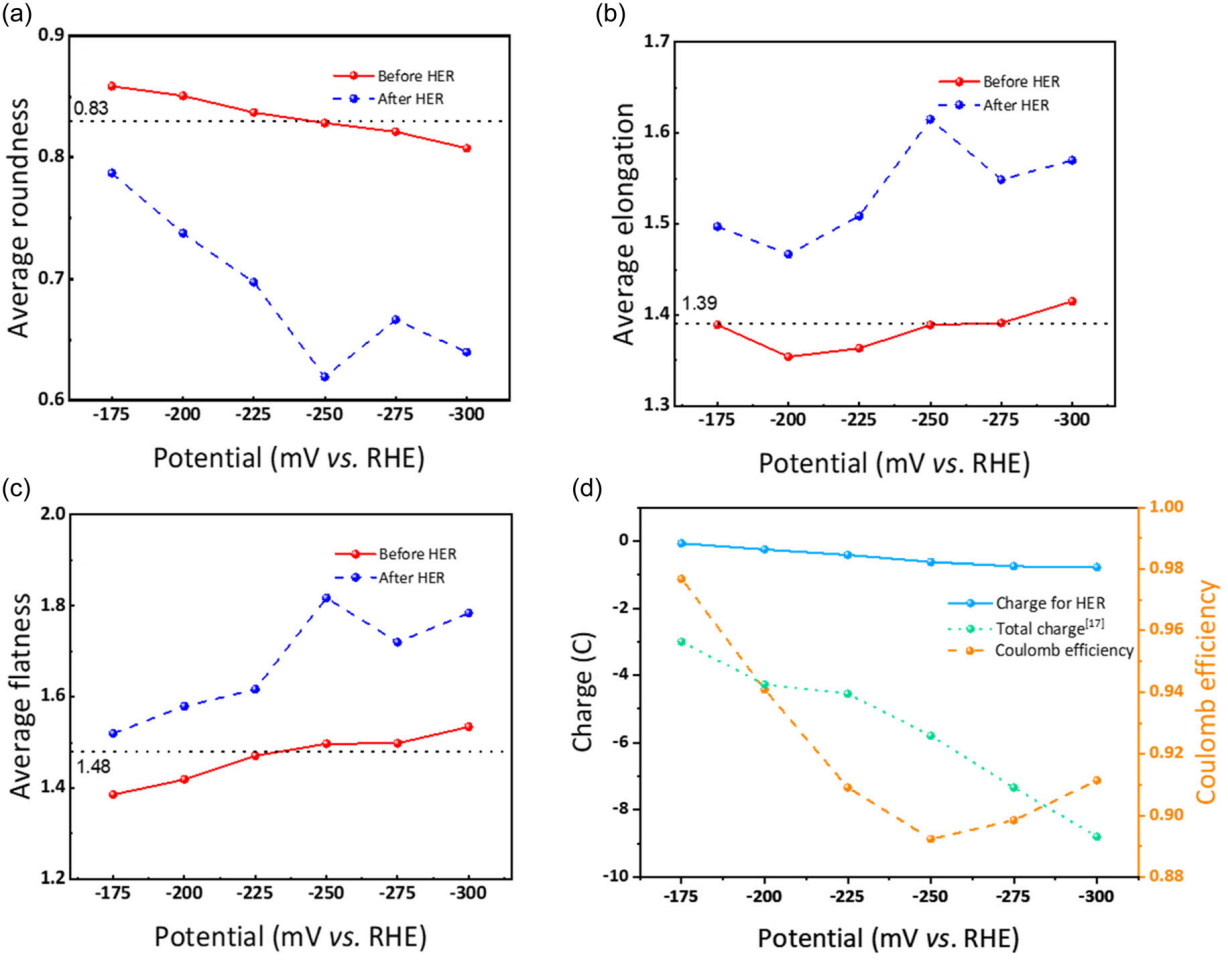
Potential‐dependent average values of the characteristic bubble shape parameters, including a) the roundness, b) the elongation, c) the flatness, and d) total integrated charge from the chronoamperogram in Ref. [17] HER‐related charge estimated from bubble volume, and the resulting coulomb efficiency for negative half‐cell.

In addition to the effect of the working electrode potential on the bubble sizes and the related bubble volume, we also investigated the differences in bubble shapes at the applied potentials. First, we determined the average values of the bubble shape characteristic parameters—roundness, elongation, and flatness—in each experiment (**Figure** **7**a–c). Furthermore, we show the corresponding coulomb efficiency during operation time.

Figure 7a–c displays the experiments’ average roundness, elongation, and flatness values at different working electrode potentials. Before the HER (red line), the bubble shapes are quite similar across all potentials, with average values of roundness at ≈0.83, elongation at around 1.39, and flatness at about 1.48. These values represent the average bubble characteristics from all experiments conducted at different voltages before HER. When the reaction finished, the bubbles in the electrode became more irregular overall due to the hydrogen evolution, as observed in the experiments of the previous part. The average shape characteristics of the bubbles in the electrode follow a distinct trend. At more negative electrode potential, the average roundness of the bubbles gradually decreases while the elongation and flatness increase. This shows that more HER activity leads to the formation of less regular bubbles caused by bubble growth in the inhomogeneous fiber network or bubble coalescence. Furthermore, the higher bubble volume also implies that more bubbles are generated or pushed closer to the borders and edges, restricting their expansion in these directions. Interestingly, the measurement at −250 mV slightly deviates from the trend of the characteristic shape parameters. The average bubble shape turns out to be less round, more elongated, and flatter than the nearby electrode potentials applied despite no problems occurring during the electrochemical measurements. Due to the limited number of experiments, we cannot clearly state if this measurement was an outlier or if there is a significant reason for interrupting the trend. Since the standard equilibrium potential of the vanadium(III) to vanadium(II) reduction reaction is at −255 mV versus SHE,^[^ ^28^ ^]^ this reaction might cause the observed irregularity. Initially, vanadium(IV) ions are reduced to vanadium(III) ions, which are further reduced to vanadium (II) if the applied potential is negative enough. Thus, −250 mV might be a point at which the vanadium(III)/vanadium(II) redox reaction starts to compete with the HER. Figure 7d further supports this interpretation: The coulomb efficiency curve exhibits a turning point at −250 mV, indicating that part of the charge is consumed by the V(III)/V(II) redox couple rather than by HER, which in turn reduces bubble formation. Although we cannot conclusively determine whether this point is a statistical outlier or a systematic shift due to the limited number of experiments, these findings suggest that the initiation of the V(III)/V(II) reaction around −250 mV diminishes HER activity, influencing both bubble morphology and the resulting coulomb efficiency.

### 2.3 Relationship between the Bubble Shape and Size

**Figure 8:**
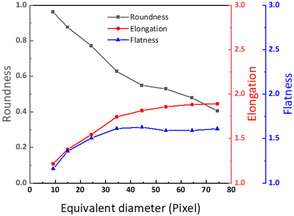
Relationship between the bubble size (given by the equivalent diameter) and the characteristic bubble shape parameters: roundness, elongation, and flatness.

This final section combines the previous analyses by showing the relationship between the bubbles’ size and shape. Due to the limited number of bubbles within a single experiment, we pooled all bubble data from all applied potentials before and after triggering the HER to finalize the study. To reduce the disturbance of the statistical law by extreme cases, the smallest two and the largest two bubbles were excluded from the analysis. **Figure** **8** shows the statistical relationship between the bubble size and the characteristic shape parameters. In general, the shape of a bubble becomes gradually more irregular as its volume increases, i.e., its roundness decreases, and elongation and flatness become larger at higher bubble volumes. The shape changes are more profound at smaller bubble sizes and become smaller when the bubble size reaches a certain value (equivalent diameter of around 320 μm in this experiment). The elongation and flatness remain very constant at larger sizes, with values of around 1.8 and 1.6, respectively. This shows that smaller bubbles, typically generated at low HER activity, tend to be rounder. In contrast, bubble growth and coalescence in the disordered fiber structure lead to more irregular bubble shapes for larger bubbles.

## 3 Conclusion

In this study, we employed high‐resolution synchrotron X‐ray tomography in combination with deep learning‐based image segmentation to systematically analyze hydrogen bubble evolution within VRFB electrodes under different applied potentials. Our quantitative analysis revealed that increased HER activity leads to the formation of larger, more irregular bubbles, which can impede electrolyte transport and reduce the effective electrochemical active area.

Importantly, our findings offer practical guidance for the optimization of VRFBs. First, the detailed characterization of bubble morphology underscores the significant influence of electrode microstructure on bubble retention. Optimizing the electrode design—by improving pore connectivity and tailoring fiber wettability through targeted surface treatments—may facilitate bubble release and promote a more uniform electrolyte distribution. Such modifications are anticipated to minimize bubble‐induced blockage, thereby preserving the effective reaction area and enhancing overall battery performance.

Second, our study suggests that operational strategies can be refined to mitigate the adverse effects of HER. For example, implementing an intermittent or pulsatile pumping strategy could periodically increase electrolyte flow to sweep away accumulated bubbles, thus preventing flow choking and ensuring efficient mass transport. Future research, including numerical simulations such as the Lattice Boltzmann Method, will further elucidate the interplay between bubble dynamics and electrode microstructure. This, in turn, will support the development of semi‐empirical models to guide the optimization of both electrode design and operating conditions.

In summary, our work not only advances the understanding of bubble evolution in VRFB electrodes but also provides concrete directions for optimizing electrode structure and operational strategies. These insights pave the way for enhanced battery performance and extended cycle life in practical applications.

## 4 Methodology

### 4.1 Experiment

A synchrotron‐adapted VRFB cell, previously designed by our group,^[^ ^18^ ^]^ was used to observe gas bubbles in a carbon‐felt electrode. A commercial graphitized carbon felt (SIGRACELLGFD 4.6 EA, SGL Carbon) based on a polyacrylonitrile (PAN) precursor was selected as the electrode and thermally treated according to a standard procedure at 400 °C for 25 h.^[^ ^29^, ^30^, ^31^ ^]^

The vanadium(IV) electrolyte used in the experiments was prepared by dissolving 0.1 M VOSO4 (vanadyl sulfate hydrate, 99.9% metal‐based, Thermo Fisher) in 2 M H_2_SO_4_ (96%, Suprapur, Merck). Since the electrolyte storage reservoir is significantly larger than the electrode volume and the reaction time is short, we assume that the electrolyte's oxidation state remains constant during the experiments.

Synchrotron X‐ray tomography experiments were used to image gas bubbles in the electrode of a VRFB half‐cell, analogously to a previous study by Köble et al.^[^ ^19^ ^]^ These include remaining air residues from incomplete electrolyte filling and hydrogen gas generated by applying potentials below 0 V versus reversible hydrogen electrode (RHE). All applied potentials in this work are presented versus the RHE potential. The measurements were performed with a white beam delivered from a superconducting wiggler located at the IMAGE beamline of the KIT Light Source. Filtered with 6 mm pyrolytic graphite, the spectrum has a photon flux density of 2.7 · 10^13^ ph s^−1^ mm^−2^ at the sample position and a wide energy distribution centered at 14.5 keV. To exploit the absorption and phase contrast, the wavefield transmitted through the sample was free space propagating 40 cm from the sample exit plane to the image plane before being detected by an X‐ray detector. The detector provided a sufficiently small effective pixel size of 4.5 μm.^[^ ^18^, ^32^ ^]^ The spatial resolution limit of the detection system was 9 μm.

Tomography scans of the electrode were conducted before and after the electrolyte injection by acquiring 2000 projections at 200 fps. For each tomography experiment, dark field and flat field images were recorded to subtract the thermal noise from each projection and correct the pixels’ different sensitivity to the impinging light.

The distribution of bubbles generated by the HER was investigated at various negative working electrode potentials in a three‐electrode setup, and the shape and size of the bubbles were characterized in detail. An initial reference tomography scan of the working electrode was performed at stopped electrolyte flow to image the distribution of gas bubbles remaining in the electrode after a period of electrolyte flow. After triggering the reactions at the working electrode (vanadium reduction reaction and HER) by applying a negative potential, a second tomogram was conducted to obtain a gas bubble distribution affected by the HER. The experiment was repeated for working electrode potentials between −175 and −300 mV.

### 4.2 Image Processing

Tomography scans were performed over 180° and then reconstructed using the Tofu image processing toolkit, to get the 3D volume of bubbles in the electrode.^[^ ^33^ ^]^ To better distinguish the voids from the remaining components, the 3D reconstruction was performed with phase retrieval.^[^ ^34^ ^]^

To standardize and facilitate image segmentation, a sufficient number of images were annotated manually to train a 2D U‐Net segmentation model with a ResNet‐34 backbone.^[^ ^35^, ^36^ ^]^ Subsequently, this model was employed to identify and label the bubbles, the membrane, and the gaskets around the membrane for the remaining experimental data. The results obtained from the deep learning model were thoroughly examined and manually aligned when required. An accurate mask of the electrode volume is essential for further analysis since the uneven membrane and the gaskets define the border of the noncuboid electrode shape.

The overall volume fraction of bubbles within the electrode was determined by calculating the ratio of the bubble volume to the electrode volume, both measured in voxels. This calculation was applied to all tomograms, enabling a comprehensive comparison between the initial and bubble distributions after the HER.

### 4.3 Bubble Characterization

After segmenting the detected bubbles in the working electrode using the deep learning model, the Insight Segmentation and Registration Toolkit (ITK) was used to analyze the bubbles’ sizes, shapes, and statistical distributions.^[^ ^37^ ^]^ The bubble shape was characterized in terms of roundness, elongation, and flatness.^[^ ^38^ ^]^

For calculation efficiency, consistency, and comparability, the bubble's equivalent radius req and boundary length *P* were used to determine the roundness *Rd*. The equivalent radius req was calculated from the actual volume *V* of the respective bubble, representing the total number of voxels comprising the bubble. For the calculation, it was assumed that the respective bubble was a perfect sphere with the same volume, *V*, and *r*_eq_ can be calculated from the volume formula of a sphere according to Equation (1).

(1) $$r_{\text{eq}}=\sqrt[{3}]{\left(\frac{3V}{4\pi }\right)}$$

The bubble's boundary length *P* corresponds to the estimated bubble's perimeter in a plane determined by its principal axis direction. In a 3D image, the boundaries of bubbles are usually composed of discrete pixels. ITK uses a method based on intercept counts (also known as discretized boundary counts) to estimate the boundary length *P* of bubbles. This method estimates the perimeter of the boundary by scanning the volume data, counting the number of planes or lines intersecting the bubble boundary, and multiplying by the image's pixel pitch. While this method is not an exact perimeter calculation, it can provide a measure of the complexity of the bubble shape. Finally, the respective bubble's roundness is calculated by dividing its equivalent circumference by the boundary length.

(2) $$\text{Rd}=\frac{2\pi \times r_{\text{eq}}}{P}$$

Elongation and flatness referred to the size difference of the bubble in the direction of the three principal axes. First, the centroid of the bubble, described by the coordinates *x*_c_, *y*_c_, and *z*_c_, should be determined by calculating its first moment (Equation (3)–(5)). Then, the second covariance matrix of the bubble was calculated, which describes the distribution of bubbles in the three principal axis directions.

(3) $$x_{c}=\frac{\sum xm\left(x,y,z\right)}{\sum m\left(x,y,z\right)}$$

(4) $$y_{c}=\frac{\sum ym\left(x,y,z\right)}{\sum m\left(x,y,z\right)}$$

(5) $$z_{c}=\frac{\sum zm\left(x,y,z\right)}{\sum m\left(x,y,z\right)}$$

(6) $$A^{T}A\xi =\lambda \xi$$

where *m* is the local binary value of the segmented images, matrix A describes the position of all voxels relative to the centroid point and *A* ^T^ denotes the transpose of matrix *A*. Using the centered second‐order matrix, the principal axis and its length were obtained by computing the eigenvalues and eigenvectors (Equation (6)). The eigenvalue matrix *λ* and eigenvector matrix *ξ* were obtained by performing eigenvalue decomposition on the centered second‐order matrix. The eigenvalues were sorted from low to high (*λ*_1_, *λ*_2_, and *λ*_3_) and associated with the corresponding eigenvectors to obtain the three principal axes. Thereby, the axis length is reciprocal to the eigenvalue, which implies that the longest axis, herein referred to as the main axis, corresponds to the lowest eigenvalue *λ*_1_. The elongation is calculated from the ratio of the other two axes’ eigenvalues (*λ*_2_ and *λ*_3_), whereas the flatness is determined from the ratio of the eigenvalue *λ*_2_ and the main axis’ eigenvalue *λ*_1_,

(7) $$\text{Elongation}=\sqrt{\frac{\lambda_{3}}{\lambda_{2}}}$$

(8) $$\text{Flatness}=\sqrt{\frac{\lambda_{2}}{\lambda_{1}}}$$

**Figure 9:**
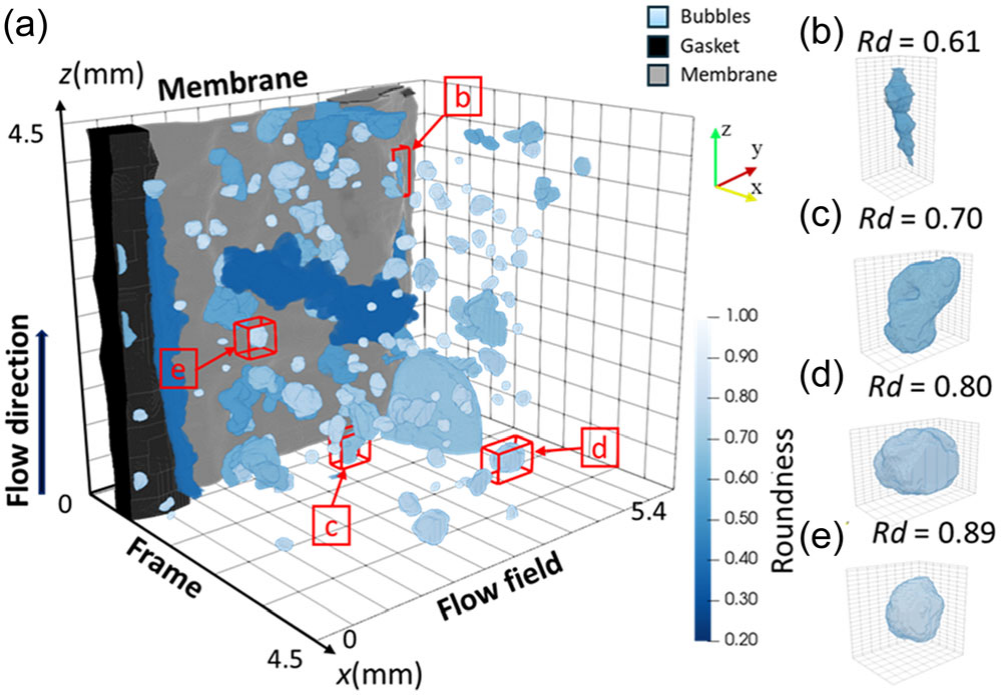
a) Exemplary display of the bubble distribution in the electrode before the HER experiment at −250 mV colored according to the bubbles’ roundness b) to e) representative individual bubbles extracted from (a) to showcase different shapes and their corresponding roundness value: b) Rd = 0.61, c) Rd = 0.70, d) Rd = 0.80, e) Rd = 0.89.

**Figure** **9**a shows an example of a 3D visualization of a segmented bubble distribution in the electrode. The size of the visualization area is 600 * 500 * 500 voxels. The electrode volume is confined by two frames (along the plane *yz*), the membrane (along the plane *xz* in the back) and the flow field (along the plane *xz* in the front). The electrolyte flow proceeds along the *z*‐axis. In this specific example, the bubbles’ colors correlate with their calculated roundness. Four individual bubbles with distinct shapes were extracted from the 3D distribution and enlarged to a similar display size (Figure 9b–e). These examples showcase specific roundness, flatness, and elongation values for the reader's reference, determined from the above calculations. Their detailed shape characteristics are provided in **Table** **1**. Note that the same color code will be used in this article's later section to highlight the bubbles’ geometrical properties and the respective distribution in the electrode volume.

**Table 1:** Shape characteristic values of the four extracted bubbles presented in Figure 9.

	Volume (voxels)	Elongation	Flatness	Roundness
b)	3969	2.13	2.06	0.61
c)	15 036	1.99	1.49	0.70
d)	33 147	1.57	1.34	0.80
e)	9395	1.10	1.31	0.89

## Conflict of Interest

The authors declare no conflict of interest.

## Author Contributions

**Kangjun Duan**: data curation (lead); formal analysis (lead); investigation (equal); methodology (equal); visualization (lead); and writing—original draft (lead). **Kerstin Köble**: data curation (equal); formal analysis (supporting); investigation (lead); visualization (supporting); and writing—review editing (lead); **Alexey Ershov**: data curation (lead); formal analysis (lead); methodology (lead); validation (lead); visualization (equal); and writing—review editing (supporting). **Monja Schilling**: data curation (equal); formal analysis (equal); investigation (equal); methodology (equal); and writing—review editing (equal). **Alexander Rampf**: data curation (supporting); formal analysis (supporting); investigation (supporting); writing—review editing (supporting); and writing—review editing (equal). **Angelica Cecilia**: investigation (equal) and methodology (equal); **Marcus Zuber**: investigation (equal); methodology (equal); and writing—review editing (supporting). **Tilo Baumbach**: funding acquisition (equal); project administration (equal); resources (lead); supervision (equal); and writing—review editing (supporting). **Pang‐chieh Sui**: funding acquisition (equal); investigation (equal); project administration (equal); supervision (equal); and writing—review editing (supporting). **Roswitha Zeis**: conceptualization (lead); funding acquisition (equal); investigation (lead); methodology (lead), project administration (lead); and writing—review editing (lead).

## Supporting information

Supplementary Material

## Data Availability

The data that support the findings of this study are available from the corresponding author upon reasonable request.
