# Supplementary material for: Investigating Bubble Formation and Evolution in Vanadium Redox Flow Batteries via Synchrotron X‐Ray Imaging
Source: ChemSusChem. 2025 Apr 24;18(13):e202500282. doi: 10.1002/cssc.202500282 (PMC12232082; doi:10.1002/cssc.202500282)
Supplement: Supplementary file 1 — Supplementary Material [file CSSC-18-e202500282-s001.pdf]

Supporting Information

Bubble characterization

Figure S1 displays the bubble size distribution analysis performed analogously to the data presented in Figure 1. A comparison of Figure S1(a) with Figure 1(a) shows that the HER at −300mV is significantly more intense and there are more large-sized bubbles generated. The number of bubbles whose equivalent diameter is below 40 pixels (360 µm) is significantly reduced after the HER period, while the number of larger bubbles remains quite similar up to a size of 80 pixels (720 µm). However, several super-sized bubbles appear with the maximum equivalent diameter reaching a value of 240 pixels (2160 µm). Due to the more negative potential of the working electrode at −300 mV, the HER reaction becomes more vigorous. In this case, it is difficult to have new independent small bubbles since they will grow and coalesce rapidly. This results in a significant reduction in the overall number of gas bubbles within the electrode and the formation of several major super-sized bubbles. These observations can be seen clearly in the cumulative bubble volume curve presented in Figure S1(b). In the region of small bubbles, the cumulative volume after the HER is lower than before the HER. Furthermore, it is notable that most volume is contributed by large super-sized bubbles. Figure S1(c) shows how much the different bubble sizes contribute to the overall bubble volume. Compared to the measurement at −200 mV, bigger bubbles remain in the electrode from the previous measurement before the HER is triggered. However, after the HER period, the two largest bubbles make up more than 75% of the overall bubble volume. Such large bubbles are undesirable since they inhibit electrolyte transport and distribution in the electrode; thus, decreasing the accessible active area and impairing the overall battery performance.

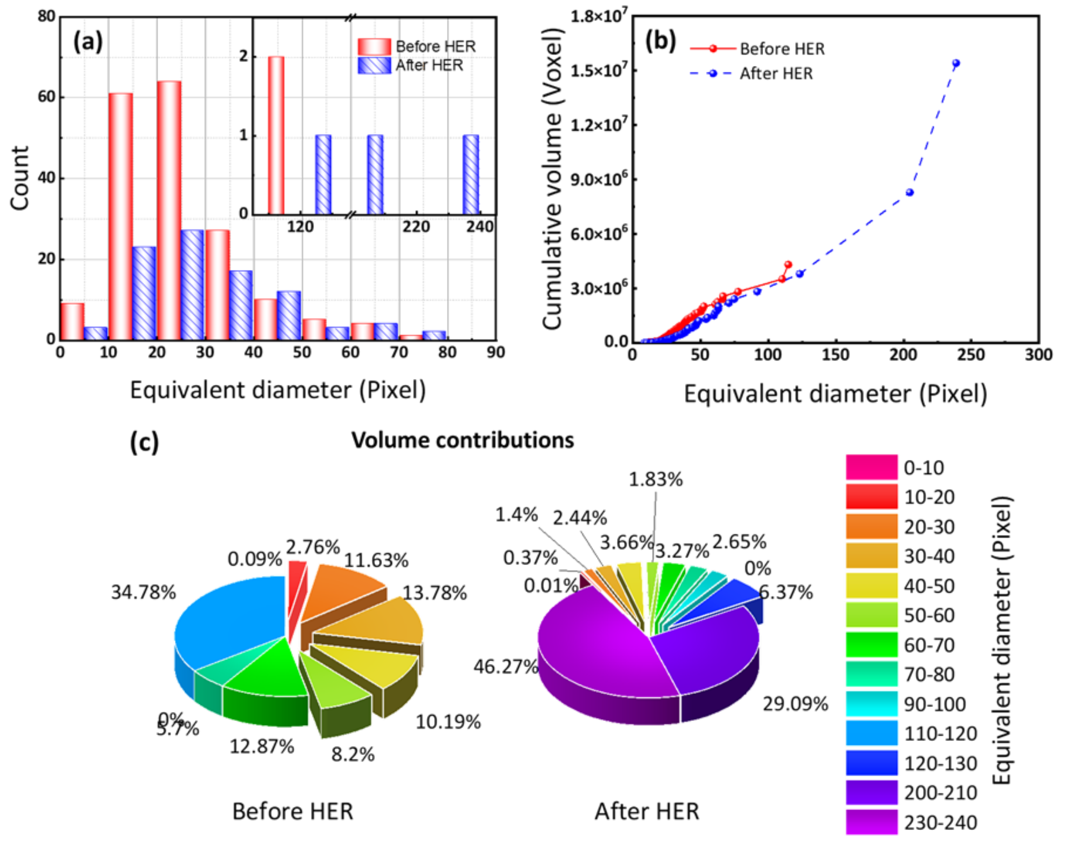

Figure S1. Comparison of the bubble sizes and their contribution to the cumulative bubble volume before and after the HER period at −300 mV: (a) Size histograms; (b) cumulative bubble volumes as a function of equivalent diameter, and (c) volume contributions of different bubble sizes.

Figure S2 displays the bubble shape analysis performed analogously to the data presented in Figure 4. The observations at the two different electrode potentials are quite similar displaying more irregular bubble shapes after the HER. At −300 mV, the average roundness decreases by 21% and the average elongation and flatness increases by 11% and 16% respectively. Overall, the average bubble roundness at −300 mV is lower compared to −200 mV and the average elongation and flatness are slightly higher. In Figure S2(b), a sub-peak emerges in the roundness frequency distribution within the 0.5-0.6 interval, which shifts to slightly lower values compared to the measurement at −200 mV. The exponential decrease in elongation and flatness is similar to the measurement at −200 mV.

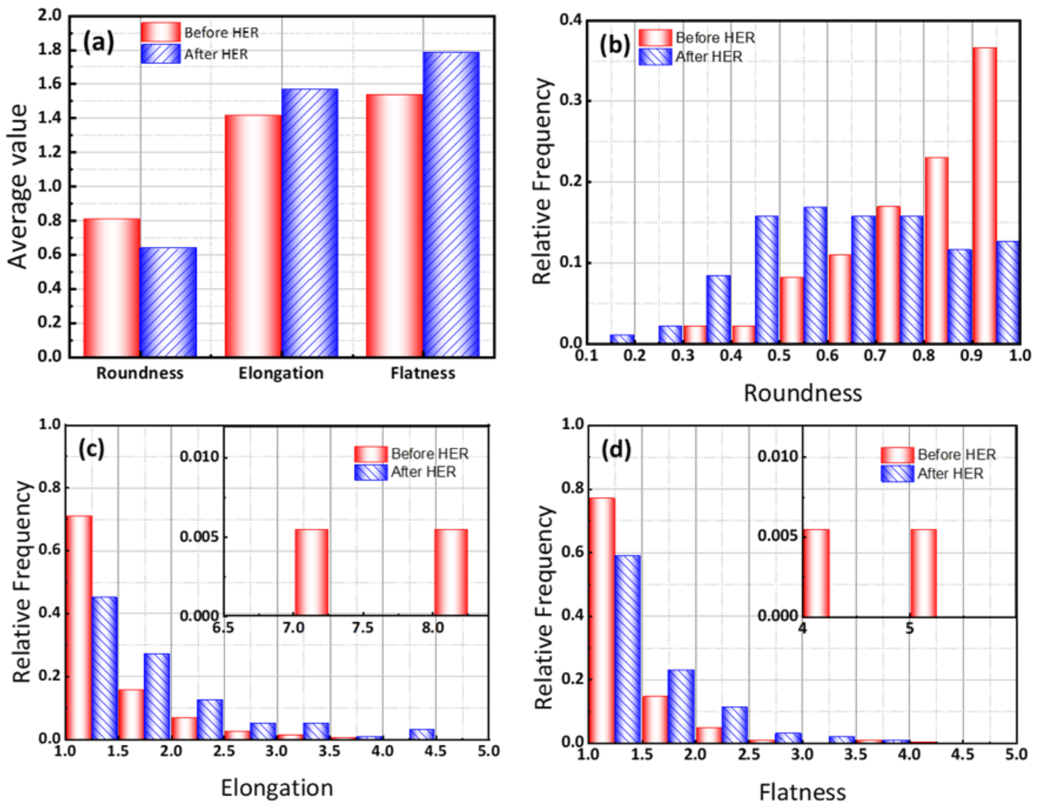

Figure S2. Comparison of the bubble shape statistics before and after the HER period at −300 mV: (a) Average shape characteristics, (b) roundness histograms, (c) elongation histograms, and (d) flatness histograms.

Figure S3 displays the 3D bubble visualization of the different characteristic shape parameters analogously to the displays in Figure 3. During the HER at −300 mV, more gas is generated in the edge region of the electrode. Unlike the separate regular bubbles observed at −200 mV, the bubbles accumulate and fuse at the edge at the more negative potential, forming a continuously expanding gas path along the surrounding frame.

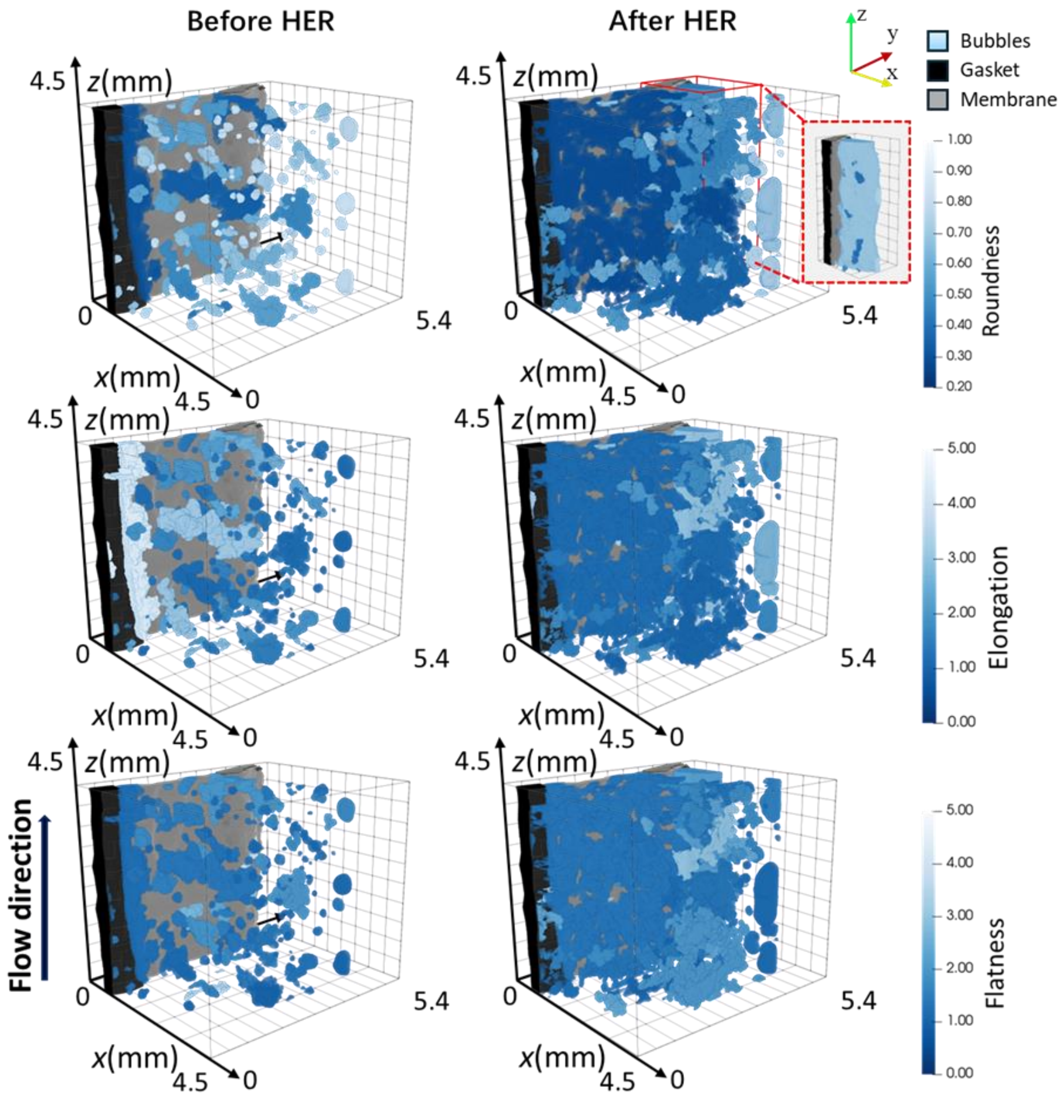

Figure S2. Comparison of the 3D visualization distributions of the bubble shape characteristics for the experiment at −300 mV before and after the HER: (a) Roundness distribution, (b) elongation distribution, and (c) flatness distribution.

Displacement of the membrane

The assembly stress of the battery will cause the membrane to deform. Figure S4 displays the displacement of the membrane in a through-plane direction. The line connecting the two extreme points on both sides of the membrane is used as the baseline.

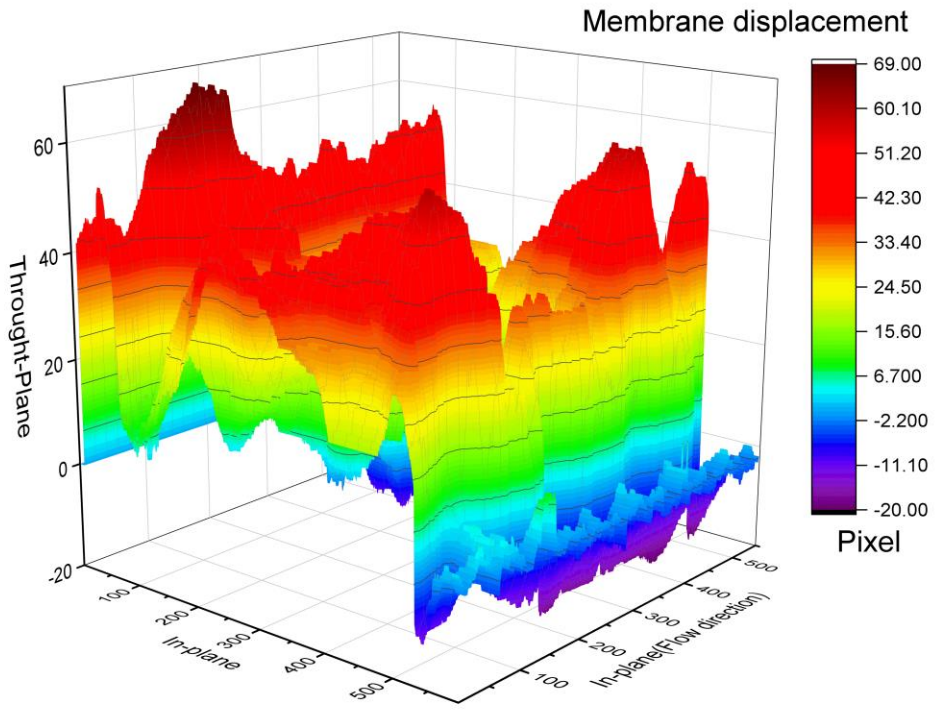

Figure S4. Displacement of the membrane in a through-plane direction

Potential-dependent bubble volumes

Figure S5(a) displays the bubble volume changes at various applied potentials, which is also presented in our previous publication.[19] Figure S5(b) shows the cumulative bubble volume before the HER at various electrode potentials.

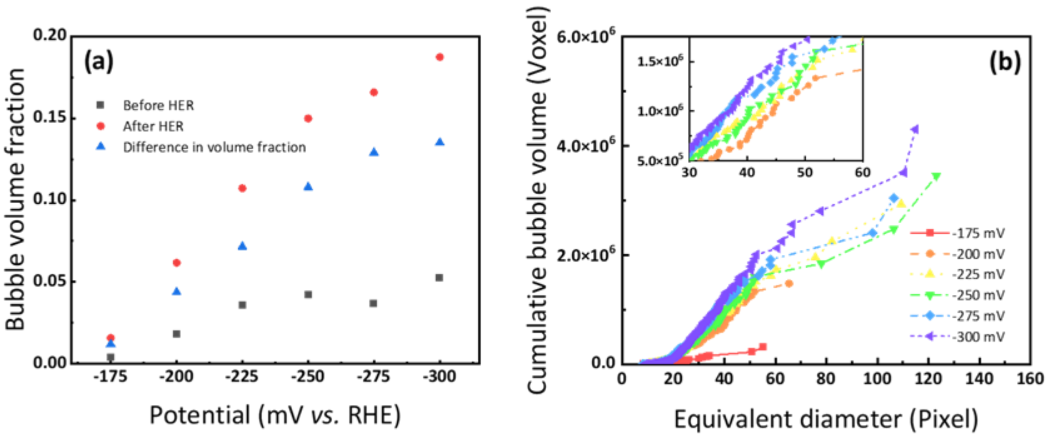

Figure S5. Bubble volume variations at different applied potentials. (a) Bubble volume fractions; (b) cumulative bubble volumes before HER.
